# Regulatory networks and molecular mechanisms underlying salt stress tolerance in rice

**DOI:** 10.3389/fpls.2026.1757448

**Published:** 2026-03-05

**Authors:** Wuyun Fang, Ali Raza, Qian Zhu, Qiming Wang, Qun Ren, Mengyang Liu, Shimei Wang, Muhammad Ahmad Hassan

**Affiliations:** 1 Rice Research Institute, Anhui Academy of Agricultural Sciences, Hefei, China; 2 Anhui Province Key Laboratory of Rice Germplasm Innovation and Molecular Improvement, Hefei, China; 3 Anhui Province Key Laboratory of Crop Integrated Pest Management, School of Plant Protection, Anhui Agricultural University, Hefei, China; 4 College of Agriculture, Anhui Science and Technology University, Chuzhou, China; 5 College of Resource and Environment, Anhui Agricultural University, Hefei, China

**Keywords:** omic techniques, proteomics, reactive oxygen species (ROS), rice, salt stress tolerance

## Abstract

Salinity and alkaline stress severely restrict rice productivity by disrupting ionic balance, generating oxidative damage, and impairing growth across developmental stages. Despite the significant advances in the salt tolerance knowledge, rice is very sensitive in contrast to other cereals, which demonstrates gaps in mechanistic understanding and breeding efficiency. This review incorporates the progress in the salt perception, signaling, and stress adaptation, and introduces limitations that slow down the practical improvement. Rice senses salinity using receptor-like kinases and Ca^2+^-dependent signaling pathway but the initial stages of the response and down-stream phosphorylation cascades have not been characterized well. The reactive oxygen species (ROS) triggered by salinity activate antioxidant mechanisms like AsA-GSH, but it is still not clear how they are spatially and organelle-specifically controlled. Proteomic analyses show extensive reorganization of proteins in signaling, cytoskeleton dynamics, metabolism and protein turnover, but most of the identified candidates have not been validated functionally. Na^+^ exclusion, vacuolar sequestration, and K^+^ retention through HKTs, NHXs, and V-ATPases are involved in ion homeostasis, but the interactions between them in tissues have not been fully understood yet. QTL studies have also reported important loci like Saltol and *qSKC1* but there are slow advances made in using them in elite cultivars. New multi-omics techniques and CRISPR-based genome editing are currently providing a chance to uncover knowledge gaps. All in all, this review presents an overall framework to develop mechanistic knowledge and speed up breeding salt-resistant varieties of rice.

## 1 Introduction

Salinity stress and alkaline stress impose distinct, though often co-occurring, physiological constraints on rice. Salinity primarily arises from excessive accumulation of Na^+^ and Cl^-^, leading to ionic toxicity, osmotic stress, and disruption of Na^+^/K^+^ homeostasis, thereby directly impairing membrane transport processes and cellular metabolism (Saleem et al., 2025). In contrast, alkaline stress is mainly associated with high soil pH, which restricts nutrient availability rather than causing direct ion toxicity (Msimbira and Smith, 2020). Under alkaline conditions, phosphorus (P) precipitates as insoluble calcium–phosphate complexes, while iron (Fe) availability declines due to reduced solubility, resulting in nutrient deficiency symptoms. Consequently, transporter responses differ markedly between these stresses: salinity tolerance predominantly depends on Na^+^ exclusion, vacuolar sequestration, and K^+^ retention mechanisms, whereas alkaline stress adaptation requires enhanced acquisition of P and Fe through specialized phosphate transporters, iron chelation systems, and pH-dependent uptake pathways (Joshi et al., 2022). Although salinity and alkalinity frequently coexist in saline–alkaline soils, their underlying physiological mechanisms and transporter behaviors are fundamentally distinct.

Rice genotypes are inherently very sensitive to saline-alkaline stress and breeding tolerant cultivars is an efficient and economical way to combat these issues. Rice genetic potential to salinity, alkalinity, drought, and cold tolerance at the seedling stage is important while, saline and/or alkaline are significantly destructive at the early seedling stage (Ren et al., 2025). Another reason is that high yielding modern varieties are comparatively more vulnerable than the traditional varieties to saline-alkaline stresses, thereby increasing the risk of major yield and production shortfalls in unfavorable years (Yang et al., 2024). In fact, in recent years, these stresses seem to worsen owing to climate change, for example, rising sea level, poor precipitation and periodic changes in the frequency and intensity of floods caused by extreme weather events (Obara et al., 2025; Praveen and Kunnampalli, 2024). By increasing of the sea level, saline water enters the fresh water farm land area and becomes saline affected. Other side, poor precipitation bound farmers to irrigate with bad quality water, sometimes saline water or water with high pH that leads to make the land alkaline. Soil minerals and weathering processes influence the soil alkalinity. Rice cultivars that are tolerant, moderately tolerant, and sensitive had yield reductions of 25%, 37%, and 68%, respectively, in saline alkali soil with a pH of 9.8 (Niu et al., 2025).

Salinity stress markedly suppresses rice performance throughout its developmental stages, leading to declines in seed germination, early seedling vigor, biomass accumulation, primary root elongation, tiller formation, and ultimately panicle mass (Saleem et al., 2025). These effects arise from reduced nutrient availability, heightened external osmotic stress, and disturbances in ion homeostasis, particularly the maintenance of cellular pH balance (Sackey et al., 2025). In order to ensure food security, rice cultivars with high tolerance to saline alkali are being developed in order to battle this stress and preserve grain output in areas affected by saline alkali. Complex molecular, physiological, and genetic pathways control saline alkali tolerance. Furthermore, different developmental phases employ various coping strategies in response to saline-alkali stress. Only a small number of rice QTLs and saline-alkaline-responsive genes have been found, and even fewer have been effectively incorporated to commercial germplasms. Hence, identification and efficient utilization of saline-alkali tolerant QTLs is crucial in rice breeding.

Because saline alkaline tolerance is governed by complex molecular, physiological, and genetic pathways, progress in breeding remains limited. Although some QTLs and stress-responsive genes have been identified, only a small number have been successfully introgressed into elite cultivars, and large gaps persist in understanding stage-specific responses and regulatory mechanisms (Sevanthi et al., 2021). Strengthening rice resilience is therefore a priority for food security, especially in regions where salinity and alkalinity are expanding.

While previous reviews have largely emphasized physiological traits, transcriptomic responses, or individual tolerance mechanisms, the present review offers a distinct integrative perspective by centering on proteome-level regulation and its coordination with signaling pathways, ion transport systems, and QTL-based genetic resources. In particular, this work highlights how post-transcriptional and post-translational regulation reshapes salt-responsive networks that are often overlooked in transcript-centric analyses. Moreover, the review explicitly addresses unresolved mechanistic bottlenecks such as organelle-specific ROS regulation, coordination among Na^+^ and K^+^ transport systems, and the limited functional validation of proteomic candidates that directly hinder translational progress.

To address these gaps, this review compiles advances in salt sensing, signaling networks, ion homeostasis, oxidative regulation, proteome remodeling, and QTL-based resources in rice. By integrating these data, the review identifies unresolved mechanistic questions and evaluates progress toward deploying molecular insights in breeding programs. By integrating mechanistic insights with breeding-relevant genetic resources, and by incorporating emerging multi-omics and genome-editing strategies, this work provides a forward-looking framework to accelerate the functional validation of candidate genes and the development of salt- and alkali-resilient rice cultivars. Salt stress response mechanisms in rice: processes of salt perception and signal transduction.

**Figure 1:**
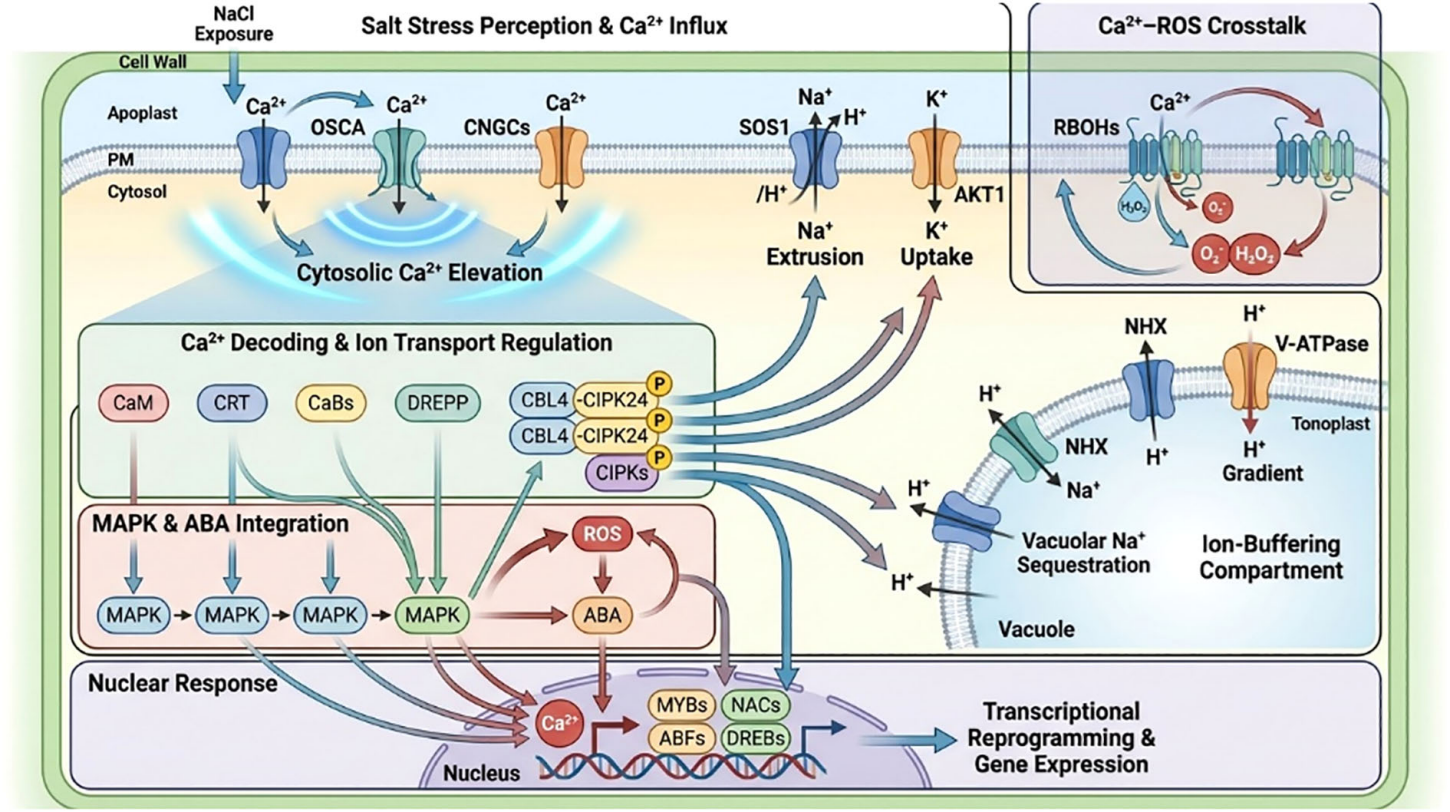
Overview of the major protein groups and pathways modulated in plant roots under salt stress. The root tip is exposed to elevated Na^+^ and Cl^-^, and the magnified region summarizes different functional categories showing upregulated (green arrows) or downregulated (red arrows) proteins. Changes are observed in carbohydrate metabolism (ADH, PDC, PGK, APDH, TPI, UGPP, UGDC, MDH, DLDH), protein processing (PPA, TcP1, TEF, RP, PD1), membrane transport (A-ATPase, REM, ANX, V-ATPase), ROS defense (POD, APX, GST, GLYI-II, TRX, SOD), and signaling (CRT, RPK, CaM, STK, DREEP, SR, GEP). Additional changes occur in cell wall polymer dynamics (actin, CCOMT, B23-chain, SAM, COMT, Tuba), transcription-related proteins (RBR, RT, SF, RTP, TrP, DRF), and protein folding components (HSP70, CPN21, CPN60, DnaK-type chaperones). Together these alterations reflect coordinated metabolic, structural, and regulatory adjustments supporting root adaptation to saline conditions.

A diverse array of receptor protein kinases (RPKs) is essential for sensing salt signals and modulating the expression of salt-responsive genes (**Figure 1**). Nonetheless, little study has been conducted on RPK activities (Abbasi and Komatsu, 2004). Proteomic studies have revealed a variety of salt-related signaling molecules present in the cytoplasm of rice root cells (Zhang et al., 2009). The frequent detection of receptor-like protein kinases (RPKs) in the apoplastic and plasma membrane proteomes of salt-treated rice roots further supports their involvement in salinity responses (Cheng et al., 2009; Zhang et al., 2009). The signal undergoes many protein changes, modifying its abundance pattern upon exposure to salt (Chitteti and Peng, 2007). Additional study is necessary to thoroughly understand this protein’s possible role in root salinity detecting. Gel-free quantification proteomic methods may be used to more effectively investigate proteins involved in salt perception or responsiveness, along with additional candidate receptors (Zhao et al., 2013).

## 2 Molecular signaling and cellular regulation under salinity

### 2.1 Calcium–mediated signaling and Ca²^+^-binding processes

Guanine (G) protein signals induce temporary elevations in cytosolic Ca^2+^ concentrations in reaction to salt stress. The brief increase is attributed to the diffusion of some internal reserves into the apoplastic region (Xiong et al., 2002). The fluctuations in cytosolic Ca^2+^ levels significantly influence root growth and development in saline environments (Munns and Tester, 2008). Calcium-binding proteins, including CaBs, DREPP, CaM, and CRT, play important roles in maintaining cytosolic Ca²^+^ homeostasis. (**Figure 1**). Proteomic analyses of rice roots under salt stress have identified several salt-responsive proteins, including CRT and calmodulin (Cheng et al., 2009; Zhao et al., 2013). Increased cytosolic Ca²^+^ activates CIPK/CBL signaling complexes. These complexes regulate important ion transporters, such as the K^+^ channel AKT1 and the Na^+^/H^+^ exchanger SOS1. Components of the MAPK cascade, including MPK4, MKK1, and MAPK3K, are also involved. They likely mediate reactive oxygen species–related signaling triggered by salt stress in rice. (**Figure 1**). The cytosolic Ca^2+^ exhibited a correlation with SOS2 and SOS3. SOS3 functions as a Ca²^+^ sensor, and salt-induced Ca²^+^ spikes activate SOS2, which in turn initiates Ca²^+^ signaling cascades that regulate multiple cellular processes in rice under salt stress. While the mechanisms of salt detection and cytosolic equilibrium of Ca^2+^ are well understood, additional investigation is required to elucidate the regulation of elemental concentrations (Ca^2+^, Na^+^). The following sections will address the roles of putative proteins, such as ANX, REM, H^+^-ATPase, and V-ATPase, which are associated with vacuolar membranes, are involved in these processes.

### 2.2 ROS scavenging and antioxidant signaling

A multitude of stress-related genes are generated by perception and transmission of signals within the cytosol (Maathuis, 2014). These genes create several essential proteins, including those implicated in root development and reactive oxygen species scavenging (Mittova et al., 2004). Reactive oxygen species (ROS) in roots can be generated through various biochemical and chemical processes. These include Haber–Weiss–Fenton reactions (Halliwell and Foyer, 1976), excess energy in the mitochondrial electron transport chain (ETC) (Møller, 2001), increased activity of NADPH oxidase at the plasma membrane (Passardi et al., 2004), and alterations in the cytosolic ascorbate–glutathione cycle (Munné-Bosch et al., 2013).

**Figure 2:**
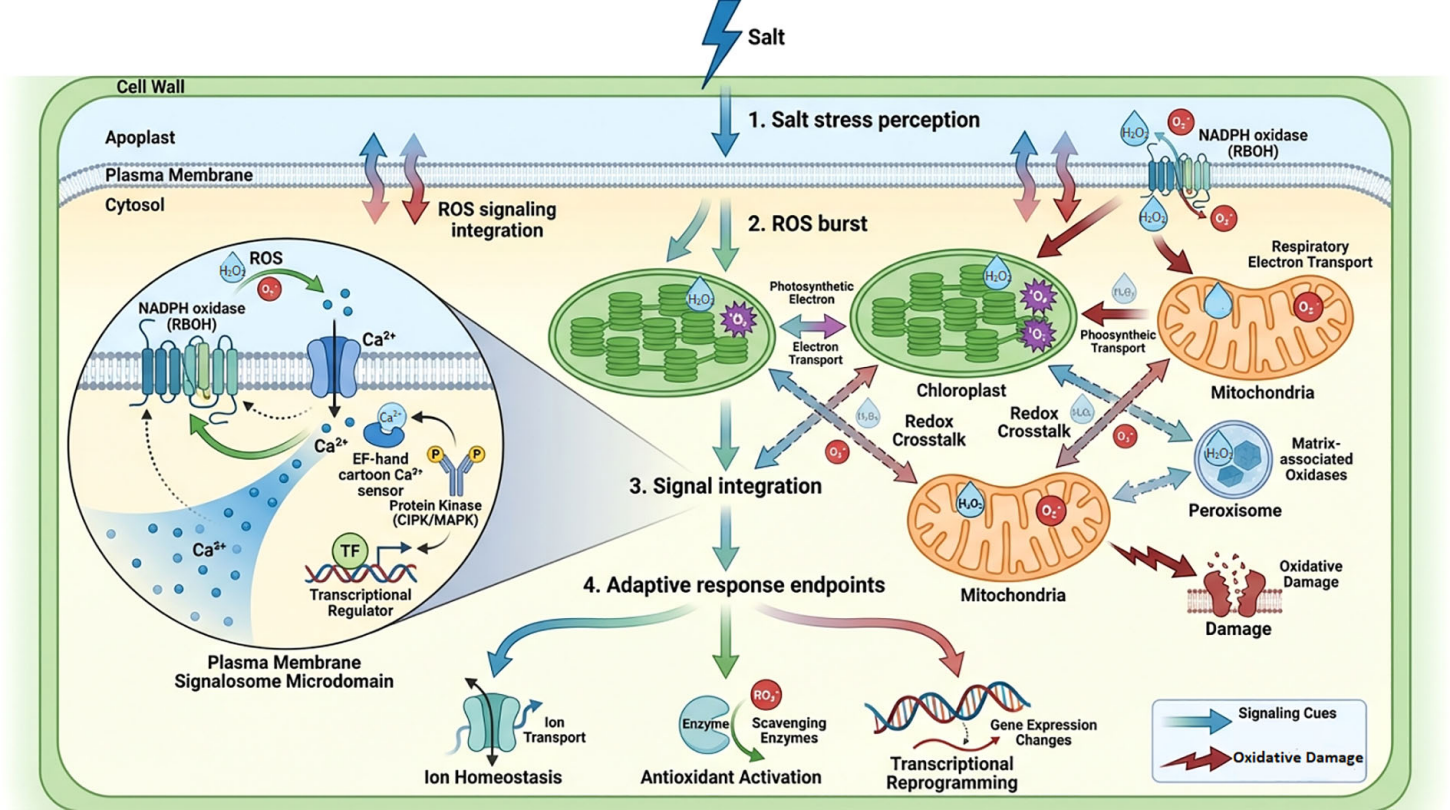
Dynamic ROS signaling, inter-organelle crosstalk, and signalosome-mediated integration during salt stress in plant cells. (1) Salt stress perception: Salt stress is perceived at the cell surface and plasma membrane, initiating early signaling events and activation of plasma membrane–localized NADPH oxidases (RBOHs). (2) ROS burst: A rapid burst of reactive oxygen species (ROS) is generated from multiple cellular compartments, including chloroplasts (photosynthetic electron transport), mitochondria (respiratory electron transport), peroxisomes (matrix-associated oxidases), the apoplast, and plasma membrane–associated RBOHs. (3) Signal integration: ROS function as signaling molecules enabling bidirectional redox crosstalk among chloroplasts, mitochondria, and peroxisomes. At specialized plasma membrane microdomains, ROS interact with Ca²^+^ channels, Ca²^+^ sensors, and protein kinases (e.g., CBL–CIPK/MAPK) to form signaling complexes (signalosomes) that integrate ROS and Ca²^+^ signals. (4) Adaptive response endpoints: Integrated signaling activates downstream adaptive responses, including ion homeostasis, antioxidant enzyme activation, and transcriptional reprogramming, while excessive ROS accumulation leads to oxidative damage. Blue/green arrows indicate signaling cues, whereas dark red jagged arrows represent oxidative stress–induced damage. SOD, superoxide dismutase; ROS, reactive oxygen species; ETC, electron transport chain; OEC, oxygen-evolving complex; H_2_O_2_, hydrogen peroxide; PS I, photosystem I; PS II, photosystem II; O_2_^-^•, superoxide anion; ¹O_2_, singlet oxygen; HO•, hydroxyl radical; XOD, xanthine oxidase; UO, urate oxidase.

The principal reactive oxygen species (ROS) in plants are H_2_O_2_, Hydroxyl radicals (•OH), superoxide anions (O_2_^-^), and singlet oxygen (¹O_2_). These chemicals are further synthesized in mitochondria, peroxisomes, chloroplasts, and other apoplastic sources (**Figure 2**). Elevated salinity lowers photosynthesis rates, which in turn increases the generation of reactive oxygen species in chloroplasts (Miller et al., 2010). In response to environmental stimuli, reactive oxygen species (ROS) function as crucial signaling molecules. NADPH oxidases, referred to as respiratory burst oxidase homologs (RBOHs), serve as critical signaling hubs in reactive oxygen species (ROS) signaling pathways. Plant RBOHs possess two Ca^2+^-binding EF-hand motifs and phosphorylation target sites in their N-terminal extension, enabling the integration of calcium signaling with ROS generation in these cells (Suzuki et al., 2011). The buildup of reactive oxygen species (ROS) due to stress activates several ROS-sensitive ion channels and disrupts ionic equilibrium inside cells, leading to the destruction of critical cellular structures. Plants have developed enzymatic and non-enzymatic systems to scavenge reactive oxygen species (ROS) in response to stress (Ahmad et al., 2010). The ascorbate glutathione recycling pathway, also known as the Halliwell Asada system, plays a key role in maintaining redox balance and is important for scavenging H_2_O_2_ in plants (Foyer and Noctor, 2011).

Genes encoding catalases (CAT), glutathione peroxidases (GPX), type III peroxidases (POD), and ascorbate peroxidases (APX) may be either upregulated or downregulated due to salt and osmotic stress (Hong et al., 2009; Mittova et al., 2004). Ascorbate peroxidase (APX), a class I heme-peroxidase enzyme, plays a major role in scavenging reactive oxygen species (ROS) by converting H_2_O_2_ into water (H_2_O) and oxygen (O_2_) (Cavalcanti et al., 2007). Rice has eight APX isoforms: two localized in the chloroplast, two in the peroxisomes, two in the cytosol, and two in the mitochondria (Teixeira et al., 2006). A substantial quantity of cytosolic APX isoforms exists in plants, and these enzymes are crucial for the leaves’ defense against abiotic stress. *OsAPX2* may modulate cytosolic H_2_O_2_ levels in response to stress, and its overexpression improved rice’s tolerance to salinity. In rice roots, NaCl stimulates the production of *OsAPX8*, which is more strongly associated with Na^+^ than with osmotic stress or Cl^-^, and this process is facilitated by an accumulation of ABA rather than H_2_O_2_ (Shigeoka and Maruta, 2014). Another important antioxidant, reduced glutathione (GSH), helps alleviate stress. Applying GSH externally increases internal GSH levels and activates enzymes such as superoxide dismutase (SOD), ascorbate peroxidase (APX), and glutathione reductase (GR), enhancing the plant’s ability to cope with salinity (Wang et al., 2014). Using NADPH, oxidized glutathione (GSSG) is reduced to GSH through the action of GR, an essential enzyme in the AsA–GSH cycle (Noctor and Foyer, 1998). Abiotic stresses linked to ABA, including salinity, strongly activate RGRC2 in rice (Kaminaka et al., 1998). One cytosolic glutathione reductase (*OsGR2*) and two chloroplastic glutathione reductases (*OsGR1* and *OsGR3*) have been identified in rice (Hong et al., 2009, 2007). In mitochondria and chloroplasts, *OsGR3* enhanced salt tolerance by modulating the redox state of GSH (Wu et al., 2015).

The AsA–GSH pathway is a metabolic route found in the mitochondria, chloroplasts, cytosol, and peroxisomes. In this pathway, ascorbate peroxidase (APX) catalyzes the reduction of H_2_O_2_ to H_2_O, using AsA as an electron donor. Other enzymes involved in this pathway include glutathione reductase (GR), dehydroascorbate reductase (DHAR), and monodehydroascorbate reductase (MDAR). Enzymatic responses to H_2_O_2_ stress can vary between plant shoots and roots (Raza et al., 2022). APX levels were found to rise under salinity in rice roots (Nam et al., 2012) as well as in leaves (Lee et al., 2001).

During salt stress, the activities of DHAR and MDAR in rice leaves were increased (Vaidyanathan et al., 2003). Overexpression of *OsDHAR1* and AeMDAR has been shown to enhance rice tolerance to salinity (Kim et al., 2014; Sultana et al., 2012). Proteomic studies also indicated elevated levels of DHAR and MDAR in rice leaves under salinity (Lee et al., 2019). Short-term exposure to 250 mM NaCl for 48 hours led to an increase in DHAR protein concentration in rice shoots (Li et al., 2011).

The activity of GR in rice varied under salt stress. Treatment of rice roots with 150 mM NaCl for 8 hours stimulated OsGR mRNA expression (Tsai et al., 2005). In contrast, proteomic analyses reported a decrease in GR levels after 3 hours of exposure to the same salt concentration (Li et al., 2010), whereas GR protein levels were elevated in roots following 7 to 48 hours of salt stress (Nam et al., 2012). These results indicate that during prolonged salt exposure (7–48 hours), GR plays a crucial role in ROS scavenging by maintaining both mRNA and protein levels in rice. This proteomic research examines the regulation of numerous enzymes associated with the AsA-GSH pathway in rice shoots, leaves or roots under varying salt stress settings. Nonetheless, transcriptional and post-transcriptional modifications caused variations in the expression and levels of these candidates. Prior research has shown that AsA-GSH-related proteins are distributed throughout several plant tissues including leaves, peroxisomes, mitochondria, cytoplasm, and roots, as well as other organelles (**Supplementary Table 1**). Currently, organelle proteomic investigations in rice subjected to salt stress remain inadequately developed. Consequently, the integrated insights of organelle proteomics may elucidate the enzymatic modifications and their pivotal functions in precisely regulated ROS scavenging networks. Moreover, all these antioxidant systems possess isoenzymes situated in various subcellular compartments, enabling precise regulation of reactive oxygen species (ROS), since specific instances, such as H_2_O_2_, exhibit signaling activities (Smirnoff and Arnaud, 2019).

### 2.3 Integrated signaling networks, tissue-specific responses, and ion–hormone crosstalk under salt stress

Rice salt stress tolerance emerges from coordinated interactions among Ca²^+^-dependent signaling, ROS-mediated regulation, ion transport processes, and hormonal control, with distinct regulatory strategies operating in roots and shoots. Salt-induced Ca²^+^ influx serves as an early signaling event that activates CBL–CIPK complexes and the SOS pathway, thereby regulating key ion transporters such as SOS1 and maintaining cytosolic Na^+^ homeostasis, particularly in root tissues that directly encounter saline conditions (Bisht et al., 2025). These Ca²^+^-mediated processes are tightly coupled with ROS signaling, as Ca²^+^-activated NADPH oxidases promote controlled ROS production that functions in downstream signal amplification.

ROS signaling acts as both a regulatory and stress-modulating component, requiring precise spatial and temporal control. Previously described, antioxidant systems such as the AsA–GSH cycle exhibit tissue- and organelle-specific regulation, reflecting differential oxidative pressures in roots and shoots. In roots, ROS signaling is closely linked to ion transport activity and Na^+^ exclusion, whereas in shoots, ROS homeostasis contributes to protection of photosynthetic machinery and redox balance under prolonged salt exposure.

Hormonal regulation integrates these signaling layers at the whole-plant level. Abscisic acid (ABA) acts as a central coordinator by modulating stress-responsive gene expression, antioxidant capacity, and stomatal conductance, thereby limiting transpirational Na^+^ transport to aerial tissues. ABA accumulation also interacts with ROS and Ca²^+^ signaling pathways, reinforcing adaptive responses under salinity (Wang X. et al., 2025). In addition to ABA, other hormones such as auxins, cytokinins, ethylene, and jasmonates influence root architecture, growth–stress trade-offs, and ion transport capacity, further shaping tissue-specific responses to salt stress (Khan, 2025).

Together, Ca²^+^/SOS signaling, ROS regulation, ion homeostasis, and hormonal networks form a highly interconnected regulatory framework that enables rice plants to perceive salt stress, partition responses between root and shoot systems, and maintain cellular and physiological homeostasis. This integrated perspective provides a mechanistic basis for understanding genotype-specific differences in salt tolerance and establishes a foundation for linking signaling pathways with downstream proteomic and multi-omics regulation.

### 2.4 Ion compartmentalization and homeostasis

The levels of elements (Na^+^, Na^+^/K^+^) in rice increase under the conditions of salt stress due to the dehydration of cells and ionic imbalance (Zhang et al., 2018). Rice has adapted various mechanisms to deal with these difficulties, and they have developed ways of ion homeostasis. Some of the mechanisms are the prevention of excessive salt intake, active extrusion of sodium through roots and the confinement of ions to particular structures within the cell (Neang et al., 2020). The sodium gets into roots through non selective cation channels (Furini and Domene, 2013). Special functions are performed by the individual transporters in the regulation of sodium flow.

*OsHKT1;5* assists in the elimination of sodium in xylem vessels, which enhances the salt tolerance (Neang et al., 2020). *OsSLAH1* is also involved in ionic transportation of sodium and chloride, which is involved in ionic balance. *OsNHX2* withdraws sodium into vacuoles, which minimizes their cytotoxic activity. *OsNHX3* works in a similar way, isolating sodium in vacuolar compartments (Neang et al., 2020). The Stress salt causes different isoforms of NHX to be expressed in roots and shoots and experimental overexpression of the affected transporter has been shown to increase salinity tolerance in rice (Neang et al., 2020; Rahman et al., 2022), and the overexpression of NHXs increases salt tolerance in rice (Neang et al., 2020). Other proteins that are found in leaf and root proteomes are essential as well. V-type proton ATPase is a proton pump, which is helpful in the storage and transport of ions into the vacuole (Damaris et al., 2016; Nohzadeh Malakshah et al., 2007), and the vacuolar K^+^ ion channel of the two-pore K^+^ (TPK) (Is V-ATPase is a proton pump and ion transporter (Damaris et al., 2016), GF14a 14-3–3 protein has a positive effect on the plasma membrane H+-ATPase, which alters the electrochemical gradient to facilitate the process of taking up sodium in the vacuoles (Nohzadeh Malakshah et al., 2007). PIP1–2 Aquaporin also assists in regulating the flux of water and ions within stressed cells.

H+-ATPase subunits have species-specific salinity responses. Subunit E is expressed in rice and alfalfa (Cheng et al., 2009; Rahman et al., 2015) and subunit A to salt stress in maize and cucumber (Du et al., 2010; Zörb et al., 2004). These proteins play a vital role in cell protection, as well as, in plant salinity tolerance. The involvement of potassium channels is also considered; TPKa helps keep potassium levels in the rice cells stable, and TPKb collaborates with it in keeping ion stability (Isayenkov et al., 2011). The combination of the activity and exaggerated expression of these various transporters and channels suggests that they are at the center of maintaining ion homeostasis under salt stress.

### 2.5 Alterations of cell wall components

Complex polysaccharides are the main component of plant cell walls, consisting of cellulose, hemicellulose, pectin, and usually comprise structural proteins and lignin (Zhang et al., 2021). Proteomic analyses have indicated that salt stress triggers certain enzymes that facilitate a change in the polysaccharide composition as well as lignin biosynthesis (**Supplementary Table 2**). In Arabidopsis (Jiang et al., 2007) and alfalfa (Rahman et al., 2015), enzymes of polysaccharide assembly are up-regulated by salinity. Also, glycosyl hydrolase family members, including GH1 and GH17 isoforms are up-regulated in Arabidopsis by salt stress, suggesting that salinity has a significant effect on cell wall carbohydrate metabolism (Jiang et al., 2007).

Salt stress also leads to the lignin deposition in the cell walls of root cells, which can have an impact on the structural integrity and mechanical properties of roots (Byrt et al., 2018). High salinity has been linked to the increment in the lignified vascular tissues and alterations in the cell wall thickness (Sánchez-Aguayo et al., 2004). The phenylalanine is converted to the monomeric units of lignin through the phenylpropanoid pathway (PPP) (Rahman et al., 2022). The initial step involved in this pathway is mediated by phenylalanine ammonia-lyase (PAL) which transforms phenylalanine into cinnamate, which is a vital reaction in the production of phenylpropanoid and lignin. Interestingly, the level of PAL decreases in rice roots in cases of stress (Li et al., 2010), and salt tolerant alfalfa genotypes induce a higher level of PAL than salt sensitive ones when subjected to salinity (Rahman et al., 2022).

Other enzymes sensitive to salt stress are caffeic acid O-methyltransferase (COMT) and the caffeoyl-CoA O-methyltransferase (CCOMT). The abundance of COMT decreases in the saline conditions in rice (Li et al., 2010) and grows in Arabidopsis (Chun et al., 2019) and alfalfa (Rahman et al., 2022). Higher COMT and CCOMT are also linked to greater tolerance to high salinity (150 mM NaCl), and speciation differences in response to the lignin pathway.

It is also shown by proteomics that methionine synthase (MS) builds up in rice subjected to salt stress (Yan et al., 2005). S-adenosylmethionine synthetase (SAMS) produces S-adenosyl-methionine (SAM) a major modulator of lignification in salient root (Sánchez-Aguayo et al., 2004). SAM was established to accumulate in lignified cells of tomato (Sánchez-Aguayo et al., 2004) and alfalfa (Rahman et al., 2015). Besides, salt-tolerant alfalfa genotypes are more active in SAMS compared to sensitive types (Rahman et al., 2015), which is confirmed by the fact that such an enzyme strengthens the process of cell wall thickening and lignification, which together contribute to the adaptation of plants to salty environments.

### 2.6 Modulation of cytoskeleton

Salt stress has extensive implications on the structural architecture in plant cells where protein filament and microtubule systems are interlinked to initiate determinant physiological activities. Actin filaments, intermediate filaments and microtubules (MTs) all play a role in cellular organization and adaptation mechanisms in salinity (Khan et al., 2023; Wang et al., 2010). The moderate levels of salinity (150 mM NaCl) could maintain microtubule assembly and bundling in Arabidopsis, but the higher levels (250 mM NaCl) initially enhanced the formation of these structures but later caused their disintegration (Wang et al., 2010). Salt exposure in rice enhanced the presence of actin and actin 7, indicating that the processes of reactive oxygen species (ROS) regulation in the root are mediated by actin rearrangements (Chitteti and Peng, 2007; Li et al., 2010; Liu S. G. et al., 2012).

**Figure 3:**
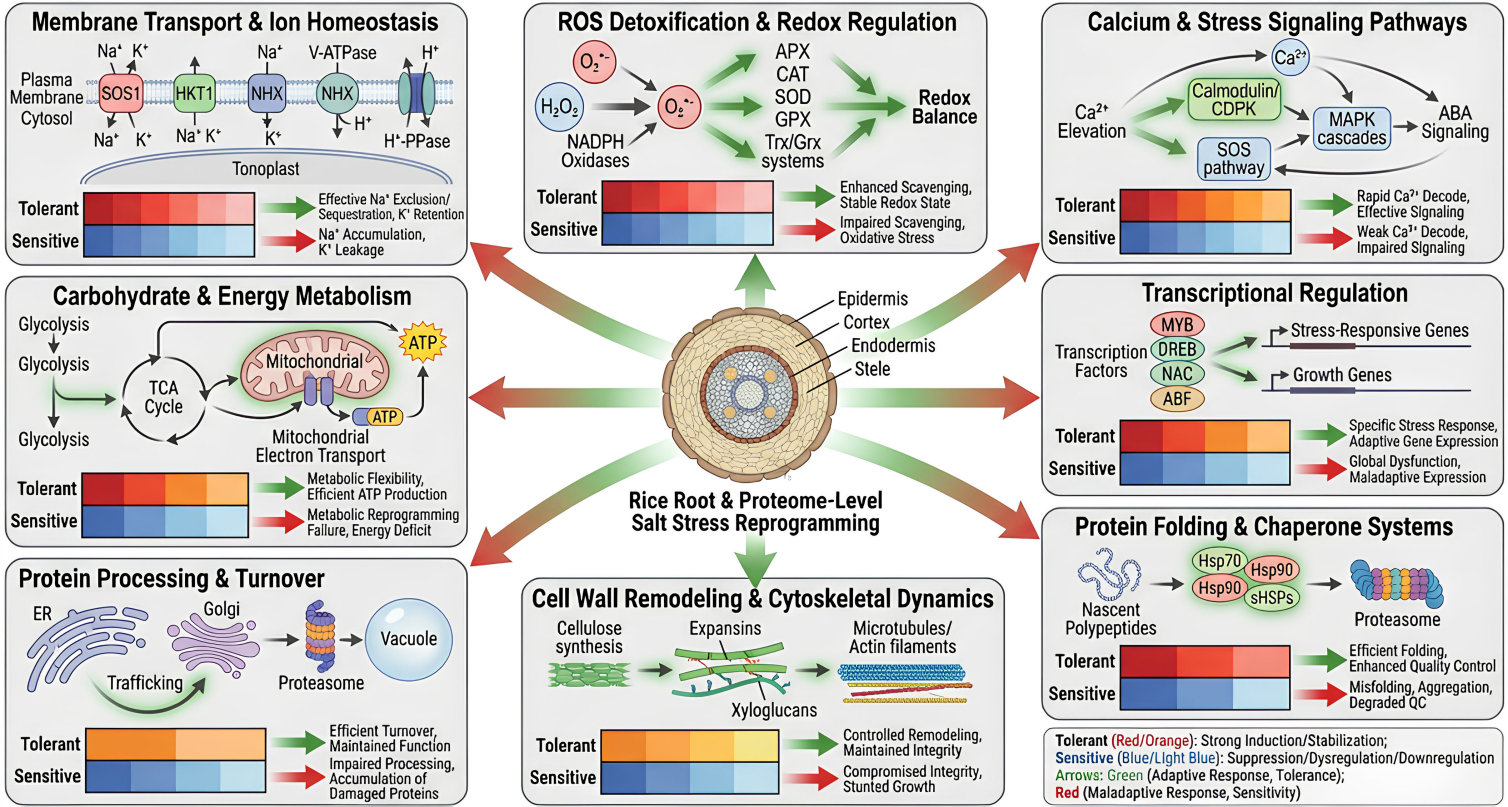
Meta-analytical overview of proteome-level reprogramming underlying salt tolerance and sensitivity in rice roots. The figure illustrates major biological processes and cellular pathways modulated in rice roots in response to salinity. Salt stress induces coordinated changes in membrane transport and ion homeostasis, including Na^+^ extrusion and vacuolar sequestration to maintain Na^+^/K^+^ balance. Reactive oxygen species (ROS) detoxification and redox regulation are activated through antioxidant enzymes and redox systems to preserve cellular homeostasis. Calcium- and stress-related signaling pathways integrate Ca²^+^ elevation, SOS signaling, MAPK cascades, and ABA-associated responses. Metabolic adjustments involve carbohydrate and energy metabolism to support ATP production and metabolic flexibility. Protein processing and turnover pathways, including trafficking and proteasomal degradation, contribute to protein quality control. Cell wall remodeling and cytoskeletal dynamics regulate structural integrity and cell expansion, while transcriptional regulation by stress-responsive transcription factors controls gene expression. Protein folding and chaperone systems assist in maintaining proteome stability under salinity. Together, these interconnected processes reflect coordinated proteome reprogramming that supports rice root adaptation to salt stress.

The actin filament organization and dynamics are regulated by actin-binding proteins (ABPs). A proteomic analysis has found a candidate ABP (AAO65861) that increases in rice roots in response to salt stress (Yan et al., 2005). Other cytoskeletal proteins such as actin 8 and tubulin b-chain were down regulated and actin a-6 levels went up (Jiang et al., 2007). There were also significant differences in cytoskeletal motor proteins: kinesins decreased, and myosins, dyneins were more expressed under salinity (Cheng et al., 2009; Chitteti and Peng, 2007) (**Figure 3**; **Supplementary Table 2**). The results show that salinity causes the rapid restructuring of the cytoskeletal elements in rice, and actin filaments and tubulin play a pivotal role in the maintenance of cellular stability and homeostasis when the condition is stressful.

## 3 Genetics of salt tolerance and QTL mapping

Transcriptional regulation of gene expression is central in ensuring that plants respond well to environmental stresses (Macrae and Long, 2012). The regulation of protein synthesis is at various phases and it includes transcriptional, post-transcriptional, translational and post-translational (Jiang et al., 2007). Proteomic studies have demonstrated that the rice subjected to salt stress has a complicated regulation network with many candidate genes having varied patterns of expression. Specifically, zinc finger proteins, basic/helix-loop-helix proteins, and reverse transcriptases are transcription factors that were stimulated by salinity in rice and other species (Amara et al., 2024; Chitteti and Peng, 2007; Sobhanian et al., 2010; Yan et al., 2005; Zhou et al., 2011).

Other enzymes that took part in the processing of RNA had changed expression as well under the salt stress. As an example, the proteins, which are involved in splicing of RNA, such as tRNA splicing protein and AAR2, were increased in rice (Nam et al., 2012; Yan et al., 2005). Similarly, the abundance of RNA-binding proteins (RBPs) was found to be altered, which indicates their role in the post-transcriptional regulation and salinity-related adaptive mechanisms (Nam et al., 2012). Translational machinery was stressed by salt, with multiple eukaryotic translation initiation factors (eIFs) including *eIF3, eIF5A, eIF5A3* and *eIF4A3* being suppressed in rice roots, suggesting that ribosome assembly at the start codons of mRNAs could have been disturbed. On the other hand, ribosomal proteins (RP S29, RP S4, RP S12-2, RP L14-like protein) and polyA-binding proteins (PABPs) and nascent polypeptide-associated complexes (NACs) were increased and reduced in response to salinity, respectively. These findings suggest that the expression of genes in the translational machinery is dynamically controlled, and *de novo* protein synthesis is coordinated by salt stress.

Crop plant traits of agronomic importance are frequently polygenic, and each of the genes is a quantitative trait locus (QTL), which has a relatively minor effect. These QTLs are important because the breeding programs to improve salinity tolerance will be identified. The tolerance to salt stresses are under the expression of various genes that participate in signal transduction, transcriptional control, ionic movements, and metabolic pathways. Recent findings in rice genomics, including availability of high-quality genome sequences, allow identifying salt-responsive genes and functional characterize proteins of signaling, ion homeostasis, and osmoregulation under salinity (Kumar et al., 2013). In addition, it has been shown that genetic variation in Saltol-QTL has been examined in 30 saline tract accessions and confirmed in 37 breeding lines with seedling-stage salinity tolerance (Chattopadhyay et al., 2014).

Quantitative trait locus (QTL) mapping of salinity tolerance in rice has been actively studied in terms of molecular marker including restriction fragment length polymorphism (RFLP), amplified fragment length polymorphism (AFLP), and microsatellites in multiple breeding populations (Bonilla et al., 2002; Thomson et al., 2010). It is important to note that Genc et al. emphasized the connection between salinity tolerance and low Na^+^ accumulation in the shoot is not always connected (Genc et al., 2007). Many reports have found QTLs associated with salt tolerance especially in seedling stage with loci found on all the 12 rice chromosomes (**Supplementary Table 1**; **Figure 2**). The highest number of QTLs is contained within chromosome 1, and the lowest number in chromosome 11.

The *Saltol* locus represents a major QTL conferring seedling-stage salinity tolerance and was mapped to the short arm of chromosome 1 between markers RM23 and RM140 explaining approximately 43% of the variation in shoot Na^+^/K^+^ ratio (Bonilla et al., 2002). A summary of major salt tolerance–associated QTLs, their chromosomal locations, effect types, and key traits is presented in **Table 1**. Subsequent studies identified Pokkali-derived alleles and the salt transporter gene *SKC1* within this region, suggesting that regulation of potassium homeostasis is a critical determinant of early-stage salt tolerance (Ren et al., 2005; Thomson et al., 2010). Additional QTLs affecting Na^+^ and K^+^ accumulation have been identified on multiple chromosomes, reflecting the coordinated control of ionic balance at the whole-plant level (Lin et al., 2004).

**Table 1:** Major QTLs and chromosomal locations.

QTL name	Trait studied	Chr.	Effect type	Remarks	Reference
*qSNC-7*	Shoot Na^+^ concentration	7	Major-effect	Explained 48.5% phenotypic variance	(Lin et al., 2004)
*qSKC-1*	Shoot K^+^ concentration	1	Major-effect	Explained 40.1% phenotypic variance	(Lin et al., 2004)
*Saltol*	Na^+^/K^+^ ratio	1	Major-effect	Flanking markers RM23, RM140	(Bonilla et al., 2002)
*qSKC1*	K^+^/Na^+^ homeostasis	1	Fine-mapped	Isolated SKC1 gene, 7.4 Kb	(Ren et al., 2005)
*qRRL2*	Relative root length	2	Novel QTL	Identified in MAGIC population	(Zhang et al., 2020)
*qSNC11*	Na^+^ concentration	11	Novel QTL	Explained 16% variance	(Wang et al., 2011)
*qSSISFH8.1*	Spikelet fertility SSI	8	Major-effect	Significant QTL under high salt stress	(Pandit et al., 2010)
*qSL.1*	Shoot length	1	Major-effect	Large effect QTL	(Mondal et al., 2022)
*qRL.2*	Root length	2	Major-effect	Large effect QTL	(Mondal et al., 2022)
*qSES3*	Seedling injury	3	Major-effect	Significant effect	(Maniruzzaman et al., 2022)
*qNa6*	Sodium concentration	6	Major-effect	Significant effect	(Maniruzzaman et al., 2022)
*qK8*	Potassium concentration	8	Major-effect	Significant effect	(Maniruzzaman et al., 2022)

Further QTL mapping studies across chromosomes 4, 6, 7, and 9 have expanded understanding of salinity-related traits, including Cl^-^ accumulation, Na^+^/K^+^ ratios, and ion distribution in leaves, particularly at the reproductive stage (Flowers et al., 2000; Koyama et al., 2001; Lin et al., 2004; Pandit et al., 2010). The major trait groups affected by salinity stress, along with their associated QTLs and mapping populations, are summarized in **Table 2**. These findings indicate that reproductive-stage tolerance involves additional genetic mechanisms linked to yield stability and fertility under stress.

**Table 2:** Key traits, major QTLs, and their effects for salt stress tolerance in rice.

Trait group	Key findings	Relevant QTLs	Population	Reference
Ion homeostasis (Na^+^, K^+^, Na^+^/K^+^ ratio)	Saltol contributes to Na^+^/K^+^ homeostasis; SKC1 causal gene	*Saltol*, *qSKC1*	Pokkali × IR29; Nona Bokra × Koshihikari	(Bonilla et al., 2002; Ghomi et al., 2013; Ren et al., 2005)
Seedling survival & injury	QTLs explain variation in seedling survival under 0.5–0.7% NaCl	*qSES3*, *qSUR1*, *qSUR5*	Akundi × BRRI dhan49	(Maniruzzaman et al., 2022)
Shoot/root length	Major QTLs identified for young seedlings	*qSL.1*, *qRL.2*	Horkuch × IR29	(Mondal et al., 2022)
Spikelet fertility & yield	QTLs for SSI, grain yield, panicle traits	*qSSISFH8.1*, *qGY3.1*	CSR27/MI48; Hasawi × BRR dhan28	(Ghomi et al., 2013; Pandit et al., 2010)
Multi-trait loci	QTLs affecting multiple traits simultaneously	*qRRL2*	MAGIC population	(Zhang et al., 2020)
Novel QTLs	Newly discovered loci under salt stress	*qSNC11*, *qRRL2*	Jiucaiqing × IR26; MAGIC	(Zhang et al., 2020)

Although phenotyping for reproductive-stage salinity tolerance is labor-intensive (Gong et al., 1999; Mohammadi et al., 2013), several studies have identified QTLs controlling tolerance during this phase, highlighting the biological relevance of stage-specific genetic regulation (Hossain et al., 2015; Reddy et al., 2017). The identification of yield-associated QTLs such as *qGY2* and germination-related loci such as *qGR6.2* further emphasizes the importance of integrating tolerance traits with productivity outcomes (Pundir et al., 2021; Zeng et al., 2021).

Bulked segregant analysis (BSA) and next-generation sequencing (NGS)–assisted mapping have greatly accelerated QTL discovery by enabling efficient identification of genomic regions associated with extreme phenotypes (Abe et al., 2012; Giovannoni et al., 1991; Michelmore et al., 1991; Schneeberger et al., 2009). QTL mapping has been extensively used in rice breeding programs, despite its complicated multigenic traits and the associated labor intensity, time consumption, and expense (Price, 2006; Salvi and Tuberosa, 2005). Consequently, the bulked segregant analysis (BSA) technique provides a direct, rapid, and effective approach to identify the genomic areas associated with markers linked to genes or quantitative trait loci (QTLs) influencing the targeted traits. This technique involves genotyping only on a pair of pooled DNA samples derived from two groups of people exhibiting severe traits (Giovannoni et al., 1991; Michelmore et al., 1991). Since 2000, high-throughput genotyping technologies using microarrays and next-generation sequencing (NGS) have seen fast advancement. These approaches, in conjunction with BSA, facilitate the discovery of several genetic markers associated with genes/QTLs of interest. The discovered genetic markers are used for the direct mapping of genes/QTLs. This has resulted in high-throughput genotyping-assisted bulk segregant analysis becoming progressively advantageous for breeders, with research utilizing this method primarily concentrating on qualitative traits (Abe et al., 2012; Schneeberger et al., 2009), whereas investigations concerning quantitative traits, especially regarding salinity tolerance during the reproductive phase, remain notably scarce.

Numerous research have documented the usage of 5–20 extreme bulks; however, the determination and standardization of the precise number of extreme bulks for QTL identification has not been thoroughly investigated. Wolyn et al. were the pioneers in proposing the eXtreme Array Mapping (XAM) methodology, using microarray-based genotyping-assisted bulk segregant analysis (BSA) for quantitative trait locus (QTL) mapping in Arabidopsis (Wolyn et al., 2004). XAM was developed as an efficient and economical approach for detecting QTLs. Takagi et al. and Yang et al. identified many QTLs associated with resistance to rice blast, grain amylase content, and germination rate at low temperatures, with NGS-assisted BSA. Despite the high-resolution genomic and mapping data offered by deep sequencing methods, the difficulty of sequencing noise due to read variations and inconsistencies in SNP density persists. Numerous statistical models have been used to mitigate the impacts of noise, notably the model described by Takagi et al., which utilized variations in allelic frequencies (Takagi et al., 2013; Yang et al., 2013). This approach has been among the most extensively used to date (Das S. et al., 2015; Lu et al., 2014). Alternative techniques include G-test-based predictions by Magwene, Willis, and Kelly, with Euclidean distance statistics to assess divergence (Magwene et al., 2011). The implementation of these approaches was expedited by the development of the QTLseqr R packages (Hill et al., 2013). A significant issue encountered in smoothed statistical analysis is its reliance on population size, QTL effects, and recombination rates. Recently, De La, Cantó, and Vigouroux provided a statistical tool (R code) to determine the locations of QTLs in a majority of F2 lines, even in the presence of low recombination rates. They also devised a simulation methodology for the identification of QTLs by constructing confidence interval statistics. This research may enhance the selection of NGS-based BSA metrics for crop enhancement (de la Fuente Cantó and Vigouroux, 2022; Mansfeld and Grumet, 2018).

Rice is vulnerable to salinity stress at several phases of its development. Tiwari et al. used bulked segregate analysis (BSA) of bi-parental recombinant inbred lines to illustrate an expedited method for QTL discovery related to salt tolerance during the reproductive phase. A 50K SNP chip was used in conjunction with BSA, identifying 34 QTL areas in the ‘CSR27/MI48’ RIL. The findings confirmed previously reported QTLs and indicated several novel ones for further investigation (Tiwari et al., 2016).

Despite these advances, the limited number of studies focused on QTL mapping and map-based cloning has constrained a comprehensive understanding of salt stress tolerance mechanisms in rice. Recent progress, however, has begun to bridge this gap. Lei et al. identified *OsSAP16*, encoding a C2H2-type zinc finger protein, as a candidate gene associated with salt tolerance using whole-genome sequencing–based QTL analysis. Elevated expression of *OsSAP16* and differential expression of neighboring genes suggest that this locus contributes to enhanced resilience under saline conditions, particularly during early developmental transitions. These findings support the potential of *qRSL7* as a valuable target for marker-assisted selection and functional validation (Lei et al., 2020).

Genetic dissection using multiparent advanced generation intercross (MAGIC) populations has further refined understanding of salt tolerance by capturing broader allelic diversity. QTLs influencing root architecture and growth responses under salinity were identified, alongside candidate transcription factors implicated in stress adaptation (Zhang et al., 2020). Complementary genome-wide association studies (GWAS) revealed loci linked to Na^+^ and K^+^ accumulation and their distribution in vegetative tissues, reinforcing the central role of ionic homeostasis as a key physiological determinant of salt tolerance (Warraich et al., 2020).

Consequently, the research elucidated the function of ionic homeostasis as a mechanism for salt tolerance. SNP genotyping was used for 18 advanced breeding lines, each possessing numerous QTLs/genes associated with stress tolerance. The lines were used for assessing yield stability and efficiency by additive main effect and multiplicative interaction (AMMI) and genotype/genotype-environment (GGE) biplot analysis (Amara et al., 2022; Debsharma et al., 2022). A reciprocal population derived from salt-tolerant Horkuch and IR29 was used to identify a significant QTL for total leaf potassium and grain weight by SNP analysis (Haque et al., 2022). Goto et al. found QTLs for the elimination of harmful Na^+^ in the leaf sheath located on chromosomes 4 and 11 (**Supplementary Table 1**), highlighting the significance of Na^+^ removal from leaf sheaths in reducing Na^+^ buildup in leaf blades (Goto et al., 2022).

To obtain the molecular basis of genetic variation in complex traits of agronomic interest, quantitative trait loci (QTLs) need to be identified which can subsequently be reduced to isolate individual genes (Fujisawa et al., 2005; Price et al., 2006). The first is the creation of near-isogenic lines (NILs) that are related, but have mutated in the genomic region of interest in the QTL. The genetic history of the frequency parent is kept in these lines permitting the impact of the targeted QTL to be analyzed as though it were a Mendelian factor. Rice genome has now been advanced and it is now possible to clone genes related to salt tolerance (Fujisawa et al., 2005; Price et al., 2006). An example of this is the *SKC1* gene which helps control potassium and sodium homeostasis of the salt-tolerant indica Nona Bokra, which was isolated by a map-based cloning strategy (Flowers et al., 2000). In other studies, seventy salt tolerance-related QTLs were discovered using recombinant inbred and doubled haploid lineages. Out of these, two genes *SKC1* and *DST* have been successfully cloned as they have a significant effect on salinity tolerance. When markers associated with these QTL region have been determined, they can be utilized in breeding studies to produce better cultivars that can be produced to perform well in saline conditions (Rasheed et al., 2022).

## 4 Proteomic mechanisms underlying salt stress tolerance in rice

One of the major constraints endangering the world’s food security is salinity in agricultural soils (Butcher et al., 2016). According to (Rahman et al., 2015), soil salinity affects 20% of irrigated soil and 6% of all land worldwide. According to (Butcher et al., 2016) and (Ahmad et al., 2016), the percentages are anticipated to quadruple by 2050. Therefore, it is very desired to use genetic engineering to increase the salt tolerance of staple food crops so that plants can adapt to and/or overcome the salinity issue. Both halophytes and glycophytes’ proteomic reactions to salt stress have been investigated (Li et al., 2011; López-Cristoffanini et al., 2021; Rahman et al., 2015). The proteome level of salt stress has been studied by plant biologists in both model plants and agricultural plants, such as rice (Ahmad et al., 2016; Liu C. W. et al., 2012; Rodriguez et al., 2021). Since salt-responsive genes and proteins are more strongly altered in roots than in shoots, plant roots are the first sensory organ to be adversely impacted by salt stress (Ahmad et al., 2016; Hasan et al., 2023). Rice under salt stress changed a number of proteins, both up-regulated and down-regulated (Chitteti and Peng, 2007; Li et al., 2011; Liu C. W. et al., 2012) (**Figure 3**).

All the overexpressed proteins were confirmed to be salt-responsive; however, some additional discovered proteins were not expressed in rice under salt stress conditions. Numerous researchers have investigated

### 4.1 Strategies of protein degradation

The ubiquitin/26S proteasome system is important in the regulation of the concentration of certain regulatory factors under stress conditions (Vierstra, 2003). Salt stress activates the modification of many factors of the proteolytic pathway. For instance, polyubiquitin, which demonstrates a change of expression, but some ubiquitin-conjugating enzymes react by becoming more active. The independent proteases also vary their activity to enable the plant to survive salinity (Zhou et al., 2010, 2012). Proteomic studies of rice have found that individual subunits of 26S proteasomes do not react in a similar manner to salt. There are those peptidases that increase in activity and there are others that show minor changes (Chitteti and Peng, 2007; Nam et al., 2012). Individual contributions of proteins that are ubiquinated have been noted in separate researches. Pyruvate is converted to phosphoenolpyruvate with the help of one enzyme which helps in energy metabolism during stress. The other one helps in development of cellulose, which is a vital aspect in ensuring the integrity of the cell wall. Meanwhile, there is a specific cyclin, which controls the cell cycle development and progression. All these proteins show specific patterns (depending on the genotype of rice), and salt-tolerant rice varieties tend to have higher abundance over the salt-sensitive ones (Liu C. W. et al., 2012). In addition to salt stress, recent studies on ubiquitination in rice immunity depict that this modification affects the stabilization of specific proteins or their degradation. The implication of such findings is that there is a broader regulatory role that spans over stress responses and defense pathways (Chen et al., 2018). Through these personal alterations, the researchers are able to point out the major regulatory proteins that play significant roles in adaptation to stress. The ubiquitination-based proteomics concepts can hence be useful in uncovering the molecular pathways that allow plants to survive salt and other environmental challenges.

## 5 Role of carbohydrate and energy metabolism related proteins, and organs development

The conversion of carbohydrates and supplying energy to the cells are vital to the continuous growth of plants, the process of development, and the ability to survive in severe conditions. These metabolic adaptations help a great deal in the ability of rice plants to survive in salty conditions (Ali et al., 2014). Proteomic studies indicate that a large proportion of energy-related pathways enzymes increase under salt stress, and these changes are most often greater in shoots than in roots (Lakra et al., 2019; López-Cristoffanini et al., 2021). Enzymes working under these conditions are involved in many biochemical pathways, both reactions leading to the TCA circle, reactions involved in electron transport and reactions needed to generate ATP, but again each element reacts separately.

A number of the enzymes in the glycolytic pathway also change in level during the experience of salinity by rice. One study reported upsurge in FBPA whereas another analysis reported upsurge in GAPDH levels. Altered activity in other independent proteomic data sets showed alterations in PGK, and individual experiments identified altered activity in ADH within similar salty conditions (Cheng et al., 2009; Chitteti and Peng, 2007; Li et al., 2010). These enzymes do not act in a homogeneous manner; however, they give evidence to the fact that energy generating pathways are rearranged when in a state of stress.

Proteomic studies have also determined salt-induced alterations in components that are associated with respiratory metabolism (**Table 3**). One type of tissue responds to pyruvate dehydrogenase, and another one to dihydrolipoamide dehydrogenase. The change in aconitate hydratase and succinyl-CoA ligase is adjusted in other organs and the malate dehydrogenase has yet another different pattern (Chitteti and Peng, 2007; Kim et al., 2005; Li et al., 2010, 2011; Nam et al., 2012). Such diffuse responses of roots, leaves and shoots stress the extensive energetic requirement of adapting to salinity.

**Table 3:** Salt-induced protein changes in rice uncovered using a comparative proteome approach.

Rice line	Tissue	Proteomic technique	Effect/Function	Effect type	Key findings	Reference
FL478 (Saltol)	Root	iTRAQ, LC-MS/MS	Osmoregulation, Protective proteins	Major effect	Roots showed strong protective response; Dehydrins and PLAT proteins contributed to salt tolerance	(López-Cristoffanini et al., 2021)
Pumba ya muwa, Moshi, Cushe, Kibawa, Basmati 217	Root	LC-MS/MS	Metabolism, Signal transduction	Moderate effect	Redox, transport, and signaling proteins enhanced salt tolerance in roots	(Damaris et al., 2016)
Nagdong	Root	2-DE, LC-MS/MS	Signal transduction, Energy metabolism	Early response	OSRK1 upregulation enhanced energy, amino acid metabolism and ROS detoxification in roots	(Nam et al., 2012)
IR64, Pokkali	Shoot	2D-DIGE	Antioxidant, Photosynthesis regulation	Major effect	Early protein response increased antioxidant activity and photosynthesis efficiency in Pokkali	(Lakra et al., 2018)
Weiguo/IR36	Shoot	KASP	Shoot growth	Major effect	Identified candidate gene OsSAP16 controlling relative shoot length under salt stress	[78]
Vytilla-4, Jhelum	Leaf	Nano-LCMS/MS	Antioxidant, Photosynthesis regulation	Moderate effect	Enhanced antioxidant enzymes and regulation of photosynthesis, amino acid metabolism, and nitrogen assimilation	(Frukh et al., 2020)
FL478, IR29	Leaf	2D, MALDI-TOF/TOF MS	Antioxidant	Major effect	Increased abundance of superoxide dismutase and ferredoxin thioredoxin reductase, boosting tolerance in FL478	(Abdollah Hosseini et al., 2015)
Kitaake, Nipponbare, Shiokari, Dular	Leaf	iTRAQ	ROS scavenging, Signal transduction	Major effect	sd58 mutant had higher ROS-scavenging activity; RGA1 regulates salt tolerance	(Peng et al., 2019)
Nipponbare (NPBA), Liangyoupeijiu (LYP9)	Leaf	iTRAQ	Ion homeostasis, Antioxidant	Major effect	Salt-tolerant LYP9 showed lower Na^+^ and Cl− accumulation and upregulated stress-responsive proteins	(Hussain et al., 2019)

Salt stress influences the electron flow and ATP production of mitochondria as well. As an example, an example group of cytochrome-c oxidase proteins becomes more abundant in rice, but decreases in Arabidopsis (Jiang et al., 2007). The subunits of the ATP synthase are also responsive across species. Salinity tolerance has been improved upon the introduction of the 6-kDa subunit of rice ATP synthase into tobacco.

The pentose phosphate pathway also plays a role in stress reactions. The independent studies show that glucose-6-phosphate dehydrogenase is modulated, and others show the changes in 6-phosphoglucono-lactonase. Individual results reveal changes in phosphogluconate dehydrogenase, and other studies again report the interference of transketolase (Bouthour et al., 2015; Kruger and Von Schaewen, 2003). Such scattered enzyme reactions depict the coordination of redox balance and precursor formation in response to stress by the pathway.

Carbohydrates are also soluble and also play significant roles. Tissues have a different response to glucose as compared to fructose, and vice versa. Other articles indicate the role of sucrose and more research contributes to the association of fructan to osmotic adaptation (Ahmad et al., 2010). A large number of proteins associated with sugar turnover are observed in shoots (Lakra et al., 2018, 2019)], and others are observed in roots (Abdollah Hosseini et al., 2015)], with more significant changes generally being detected in aerial tissues (Lakra et al., 2019). Though these metabolic adaptations do not cause salt tolerance directly, they promote downstream protective mechanisms like compatible solutes (e.g. proline and glycine betaine) accumulation, antioxidant machinery activation, and activation of salt-perceiving and salt-responding signaling pathways (Das S. et al., 2015).

## 6 Improvement of salt tolerance in rice using proteome and other omics approaches

The more recent developments in a variety of biological experiences, including large-scale data on genomes, and experiments involving cellular metabolites, cellular behavior and plant characteristics have significantly enriched our comprehension of crop reaction to stressful circumstances around them. Although transcript-level studies are still prevalent, knowledge on the basis of protein-based studies and metabolic profiling has become equally important in explaining how plants adapt to abiotic environments. Combination with these different analytical platforms has significantly enhanced the finding of breeding targets to stress-resilient varieties (Dai et al., 2022; Derbyshire et al., 2022). Here, we draw attention to the role of the protein-based studies, as well as other multi-layered strategies in biology, in advancing the research in terms of improving the rice growing and salinity resiliency via genetic engineering, Molecular Breeding, and genome editing.

Plant protein-based approaches offer a much-needed interface between gene activity and functional cellular responses as alterations in transcript abundance do not always reflect the ultimate protein expression as a result of post-translational regulation (Komatsu et al., 2009; Wang and Komatsu, 2020). This is why cellular metabolite and global protein processes analyses are invaluable to the interpretation of genome-based forecasts. Protein profiling can help scientists describe changes in protein levels caused by stress, study system-wide molecular behavior, and find interaction modules between candidate proteins (Das P. et al., 2015). These analyses have revealed that exposure to salt causes extensive protein-level responses in rice and a series of studies have mapped these responses by various proteome-oriented workflows (Wei et al., 2021).

Simultaneously, transcript signatures and metabolic pattern assessment studies have been applied to compare tolerant and sensitive cultivars, and domesticated lines and wild relatives of rice in saline environments (Tiwari et al., 2022). When such layers of evidence are integrated, one can find the same themes of molecular characteristics of salt adaptation. Altogether, these multi-tiered datasets can form a powerful basis of breeding and crop improvement initiatives that are intended to produce rice genotypes with better salt tolerance.

The development of genome-editing tools based on clustered regularly interspaced short palindromic repeats and the associated Cas proteins in recent years has enabled the introduction of new possibilities in order to modify plant genomes with unprecedented precision. These CRISPR/Cas systems are a significant breakthrough in the molecular manipulation strategy and have been used successfully in various organisms (Farhat et al., 2019). Researchers have used a construct containing Cas9 and a guide RNA to *OsRR22* in rice, in which case, the genome is transformed in transformed lines. The modified plants were significantly more resistant to saline environments than unmodified controls (Riaz et al., 2025; Zhang et al., 2019).

Even though genome editing has been discussed separately, it has some intersections with proteomics. Contemporary protein level research is beginning to make use of CRISPR/Cas technologies to model customized cellular systems, as well as to deconstruct protein-protein interactions and chromatin-tethered interactions. Regardless of this possibility, systematic application of genome editing towards proteome-oriented queries in plants has not yet been exhaustive (Dolgalev and Poverennaya, 2021). However, the advances made by CRISPR/Cas have already been useful in proteomics, and rice studies in specific have already gained more in the case of more extensive application (Zhang et al., 2019). An example would be to use quantitative proteomic profiling to initially identify proteins that are responsive to salt exposure; the candidates can be subsequently knocked out using CRISPR/Cas to determine the functional effects of the phenotype on plant performance in response to stress. By doing so, genome editing will become an effective companion of molecular breeding programs that seek to improve salinity tolerance.

Recent studies have also shown that it is possible to create new lines that are more resistant to salt by editing certain parts of the genome in rice (Khan et al., 2021). An example is the *OsqSOR1* gene which is a homologue of the *AtDRO1* gene that regulates the position of shallow root development. One nucleotide change in the third exon of *OsqSOR1* will cause an early stop codon that forms surface-oriented roots an architectural constraint that leads to enhanced seedling performance in salty environments (Kitomi et al., 2020). In yet another study, two independent CRISPR-Cas9 mutations were introduced in *OsRR22* (one was 20 bp upstream of the M16 exon and the other was 1 bp upstream of the M18 exon). These changes stimulated the growth of shoot and root biomass and leaf production under salt stress (Han et al., 2022).

Plant G-protein signaling systems are also involved in the control of stress. The null mutants that resulted after CRISPR-Cas9 disruption of *gs3* and *dep1* genes had increased salinity tolerance (Cui et al., 2020). In addition to genome editing, a number of complementary breeding systems such as genome-wide association method, genetic engineering methods, and marker-assisted selection are still going on to fasten molecular advancement in rice enhancement. Collectively, the tools provide useful channels to come up with cultivars that are capable of remaining productive despite the rising environmental staples.

## 7 Multi-omics dissection of salt stress responses in rice

### 7.1 Epigenetic regulation, DNA methylation dynamics, and stress memory under salinity

Recent (Rajkumar et al., 2020; Talarico et al., 2025). Whole-genome bisulfite sequencing analyses revealed that salinity alters cytosine methylation in CG, CHG, and CHH contexts, with pronounced changes occurring in promoter regions and transposable elements proximal to stress-responsive genes (Rajkumar et al., 2020). These methylation changes are often associated with altered chromatin accessibility and differential transcriptional responsiveness of genes involved in ion transport, hormone signaling, and oxidative stress mitigation. Importantly, salt-induced methylation patterns are not uniformly repressive; instead, both hypermethylation and hypomethylation events contribute to fine-tuning gene expression in a locus- and context-dependent manner.

Evidence further indicates that epigenetic regulation contributes to stress memory and adaptive priming (Banerjee and Roychoudhury, 2017; Rashid et al., 2022). Plants exposed to salinity display altered chromatin states that persist after stress removal, enabling faster or stronger transcriptional reactivation upon subsequent salt exposure. Such epigenetic priming has been proposed as a mechanism underlying phenotypic plasticity and potentially transgenerational inheritance of salt tolerance traits. These findings underscore that epigenomic modifications shape long-term regulatory capacity under salinity and provide a mechanistic explanation for variation in stress responses that cannot be explained by DNA sequence polymorphisms or transcript abundance alone.

Recent studies further demonstrate that epigenetic regulation directly intersects with core salt stress signaling and ion homeostasis pathways. A notable example is the dynamic DNA methylation of the promoter region of *OsHKT1;5*, a key Na^+^ transporter controlling root-to-shoot Na^+^ translocation. Salt-induced changes in promoter methylation have been shown to modulate *OsHKT1;5* expression, thereby fine-tuning Na^+^ exclusion efficiency in response to environmental conditions (Wang et al., 2020). These epigenetic modifications provide an additional regulatory layer beyond transcription factor binding and Ca²^+^/SOS signaling, enabling flexible and reversible control of ion transport under salinity.

Importantly, accumulating evidence indicates that such epigenetic states can be influenced by environmental history and, in some cases, partially transmitted to subsequent generations. This transgenerational stress memory contributes to enhanced salt responsiveness in progeny without permanent genetic alteration, highlighting the potential of epigenetic variation as a breeding-relevant trait. Integrating DNA methylation dynamics, histone modifications, and chromatin remodeling with established signaling pathways therefore offers a more complete framework for understanding salt stress adaptation and provides new opportunities for breeding strategies that exploit both genetic and epigenetic plasticity.

### 7.2 Transcriptomic reprogramming and transcription factor–centered regulatory networks

Transcriptomic profiling has revealed large-scale reprogramming of gene expression in rice exposed to salt stress, affecting pathways related to ion homeostasis, hormone signaling, redox balance, and primary metabolism. RNA-seq analyses of *OsNAC113* knockout lines demonstrated that this transcription factor regulates a broad gene network encompassing Na^+^ and K^+^ transporters, ROS-scavenging enzymes, and osmoprotectant biosynthesis pathways. Loss of *OsNAC113* function resulted in widespread transcriptional dysregulation and heightened salt sensitivity, confirming its role as a central transcriptional regulator of stress responses (Wang B. et al., 2025).

Similarly, transcriptomic dissection of the *OsMYB2–OsANT1* regulatory module revealed coordinated regulation of ABA-responsive genes and amino acid transport systems under salinity. While transcriptional activation of this module contributes to stress adaptation, these studies further demonstrated that regulation of the amino acid transporter *OsANT1* by *OsMYB2* supports metabolic balance and carbon–nitrogen allocation under stress conditions (Nie et al., 2025; Tong et al., 2025). Importantly, this regulatory mechanism contributes to the maintenance of grain yield under salinity, thereby linking transcriptional stress responses with agricultural productivity rather than stress survival alone. These findings indicate that transcriptional reprogramming must be coupled with downstream metabolic and physiological coordination to sustain yield under adverse environments.

Notably, transcriptomic changes frequently exceeded phenotypic effects, indicating buffering or modulation at post-transcriptional and post-translational levels. Collectively, these findings highlight that transcriptomics captures early regulatory signals but provides an incomplete picture of functional stress adaptation.

### 7.3 Post-transcriptional regulation mediated by small RNAs and RNA stability control

Post-transcriptional regulation, particularly via small RNAs, further refines salt stress responses by controlling transcript stability and translational efficiency. Multiple studies have identified salt-responsive microRNAs in rice that target key regulatory genes, including transcription factors, ion transporters, and antioxidant enzymes (Parmar et al., 2020; Wan et al., 2022). These microRNAs act as molecular rheostats, dampening excessive transcriptional activation and enabling rapid, reversible modulation of gene expression in response to fluctuating salt conditions.

Integration of small RNA profiling with transcriptomic data revealed frequent inverse relationships between microRNA abundance and target transcript levels, contributing to the observed uncoupling between mRNA accumulation and protein output. This layer of regulation is especially important under prolonged or fluctuating salinity, where tight control of gene dosage is required to balance growth and stress defense. These findings reinforce the necessity of incorporating RNA-level regulation into multi-omics frameworks aimed at understanding salt tolerance.

### 7.4 Functional validation through genome editing and metabolic reprogramming

Genome-editing studies have provided decisive evidence that salt tolerance in rice can be governed primarily by post-translational regulation rather than transcriptional control. A notable example is the targeted modification of the calmodulin-binding domain (CaMBD) of *OsGAD4*, a key enzyme catalyzing γ-aminobutyric acid (GABA) biosynthesis. Using CRISPR/Cas-mediated truncation of the CaMBD, this study demonstrated that Ca²^+^/calmodulin interaction acts as a negative regulatory switch controlling GAD4 enzymatic activity. Removal of this regulatory domain resulted in constitutively elevated GABA accumulation under both control and stress conditions (Akter et al., 2024).

Importantly, transcriptomic analysis showed that *OsGAD4* mRNA levels were only modestly altered, indicating that enhanced GABA production was not driven by transcriptional upregulation but by release of post-translational inhibition. Metabolomic profiling revealed significantly increased GABA levels in edited lines, which correlated with improved tolerance to salinity, drought, and flooding stresses. These phenotypes were associated with enhanced osmotic adjustment, redox balance, and stress recovery capacity, underscoring the functional importance of metabolic regulation downstream of gene expression.

Importantly, this genome-editing strategy represents a conceptual advance beyond salt-specific tolerance. The *OsGAD*-edited rice lines exhibited enhanced tolerance not only to salinity but also to drought and flooding, indicating that modulation of the GABA metabolic pathway confers broad-spectrum abiotic stress resilience (Kulsum et al., 2025). This finding highlights metabolic reprogramming as a shared buffering mechanism across multiple environmental stresses and shifts the focus from stress-specific responses toward common regulatory nodes that support plant survival and performance under diverse adverse conditions. Such multi-stress tolerance is particularly valuable in the context of climate change, where salinity often co-occurs with drought or flooding events.

This work provides a clear mechanistic link between Ca²^+^ signaling, protein-level regulation, and metabolic stress adaptation, illustrating how genome editing can be used to validate predictions emerging from multi-omics analyses. Such findings reinforce the central role of proteome-level control in shaping stress resilience and demonstrate how integrative omics approaches can inform precise genome-editing interventions for crop improvement.

### 7.5 Genome-wide association studies and multi-omics integration in rice salt stress tolerance

Genome-wide association studies (GWAS) have substantially advanced the dissection of salt stress tolerance in rice by exploiting natural genetic variation across diverse germplasm panels and developmental stages (Warraich et al., 2020). Early large-scale GWAS using a global diversity panel of over 300 *Oryza sativa* accessions under controlled salinity (50 mM NaCl) revealed that salt tolerance is highly stage-dependent, with distinct genetic architectures governing short-term (6 h), medium-term (7 d), and long-term (30 d) responses (Patishtan et al., 2018). Phenotyping based on relative growth rate and tissue Na^+^ and K^+^ concentrations demonstrated extensive genotypic variation, particularly in Na^+^ exclusion and Na^+^/K^+^ homeostasis, traits that are more mechanistically informative than growth alone. GWAS identified approximately 1,200 candidate genes, including cation transporters, transcription factors, and a notable enrichment of genes associated with the ubiquitin–proteasome pathway, suggesting that protein turnover and stability contribute significantly to phenotypic variation in salt tolerance.

Subsequent gene-based GWAS conducted at the reproductive stage further refined the genetic basis of salinity tolerance by targeting stress-responsive genes distributed across all rice chromosomes. Using a custom SNP array representing approximately 6,000 stress-related genes, association mapping in 220 rice accessions identified multiple loci significantly associated with Na^+^/K^+^ ratio, ion accumulation, and yield-related traits under saline field conditions (Kumar et al., 2015). Importantly, the well-characterized Saltol region on chromosome 1, previously linked to seedling-stage tolerance, was also detected as a major association at the reproductive stage, alongside additional loci on chromosomes 4, 6, and 7. These findings highlighted both shared and stage-specific genetic determinants of salt tolerance and underscored the quantitative and polygenic nature of the trait.

More recent GWAS focusing explicitly on reproductive-stage salinity tolerance evaluated diverse rice panels under field-level saline stress and integrated physiological, ionic, and yield-related traits (Warraich et al., 2020). Association analyses identified marker–trait associations primarily linked to Na^+^, K^+^, and Na^+^/K^+^ uptake in leaves and stems, with individual loci explaining modest proportions (approximately 5–13%) of phenotypic variance. Many associated markers were located near genes encoding transcription factors, membrane transporters, and signal transduction components, reinforcing the view that regulatory processes, rather than single structural genes, dominate natural variation in salt tolerance.

Despite the power of GWAS to identify genomic regions associated with salt tolerance, functional interpretation of association signals remains challenging due to linkage disequilibrium, small effect sizes, and strong environmental modulation. Integration of GWAS with transcriptomic, epigenomic, and proteomic datasets provides a critical pathway to resolve these limitations. For example, the enrichment of ubiquitination-related genes in GWAS intervals aligns with proteomic evidence showing salt-induced remodeling of protein abundance and stability, suggesting that post-translational regulation represents a key mechanistic layer underlying GWAS signals. Similarly, integrating ionomic phenotypes with proteome-level regulation of transporters and signaling components helps explain why transcript abundance alone often fails to predict Na^+^ exclusion efficiency or long-term tolerance.

Beyond Asian rice (*Oryza sativa*), recent studies from Europe and Africa have demonstrated that African rice (*Oryza glaberrima*) harbors distinct salt tolerance mechanisms arising from its independent domestication history and long-term adaptation to marginal and saline environments. Comparative genomics and genome-wide association analyses have identified unique loci and haplotypes in *O. glaberrima* associated with Na^+^ exclusion, root system architecture, and stress-responsive signaling pathways that are rare or absent in *O. sativa (*Mheni et al., 2025*)*. These findings indicate that salt tolerance has evolved through partially divergent genetic routes in different rice species.

Importantly, the identification and deployment of wild- and African rice–derived haplotypes provide a foundation for “smart breeding” strategies that combine GWAS-guided locus discovery with haplotype-based selection, introgression, and genome editing. Such approaches enable targeted incorporation of favorable alleles while minimizing linkage drag and yield penalties. Integrating genetic diversity from *O. glaberrima* and other wild relatives therefore expands the breeding toolbox and enhances the global relevance of salt tolerance research, particularly for saline-prone regions in Africa, Europe, and Asia.

Collectively, GWAS studies across developmental stages demonstrate that rice salt tolerance is controlled by numerous regulatory loci with context-dependent effects. Embedding GWAS results within a multi-omics framework enables prioritization of causal genes and regulatory modules, facilitates functional validation through genome editing, and enhances the translational value of association signals for breeding salt- and alkali-tolerant rice cultivars.

### 7.6 Multiomics guided salt tolerance in rice and soil sustainability

Multi-omics dissection of salt stress responses has enabled the identification of regulatory nodes, metabolic pathways, and genetic architectures that support stable rice performance under saline conditions. Deployment of salt-tolerant rice cultivars developed through these insights has implications beyond stress adaptation, extending to soil management practices and environmental sustainability. In saline and coastal paddy systems, salt-tolerant genotypes identified through transcriptomic, proteomic, and GWAS-guided approaches allow greater flexibility in irrigation and water-level management without compromising yield (Bulegeya et al., 2025; Nguyen et al., 2025).

Importantly, water management strategies strongly influence methane (CH_4_) emissions from paddy soils (Zhang et al., 2025). Rice cultivars capable of maintaining productivity under moderate salinity enable reduced flooding intensity or intermittent irrigation practices, which are known to suppress methane emissions by altering soil redox conditions and microbial activity (Khatibi et al., 2025). Thus, multi-omics–guided improvement of salt tolerance indirectly contributes to mitigation of greenhouse gas emissions from rice agroecosystems.

In addition, salt-tolerant rice varieties can facilitate the productive use and gradual rehabilitation of saline soils, reducing pressure on non-saline agricultural land and minimizing excessive chemical inputs (Saleem et al., 2025). Root ion homeostasis traits and stress-responsive metabolic pathways identified through multi-omics analyses may also influence rhizosphere processes, further linking molecular stress adaptation to soil health. Together, these considerations highlight how multi-omics–informed breeding strategies can connect molecular mechanisms of salt tolerance with sustainable soil management and climate-resilient rice production.

## 8 Future perspective

The rapid evolution of plant omics, genome editing, and the high-resolution phenotyping are changing the perception of salt tolerance in rice; nevertheless, there is still a series of scientific and translational gaps. To begin with, it is essential that initial events in salt perception, such as the biochemical characteristics and tissue selectivity of receptor-like kinases and Ca^2+^ signals, downstream phosphorylation networks, and the biochemical and molecular properties of these networks, be functionally validated further. Proteomic work has revealed hundreds of proteins responsive to salt, although most are not characterized *in vivo*, which means that integrative phosphoproteomics, targeted mutagenesis, and cell-specific protein mapping are needed to understand the mechanisms of their action. Second, the signal of reactive oxygen species (ROS) remains a challenge in the conceptual framework. Even though the pathways of antioxidants like AsA-GSH have been well elucidated, the localization of both the generation and elimination of ROS within chloroplasts, mitochondria, peroxisomes, and apoplasts remain poorly uncovered. The study of ROS-directed stress adaptation of organelles combining organelle proteomics, redox metabolomics, and bioimaging technologies would offer unparalleled understanding in the future.

Better salt tolerance will also be based on a better understanding of ion transport coordination. Although there have been advances in defining *OsHKT, OsNHX*, V-ATPase and TPK channel families, the relationships between these systems particularly in relating to varying salt regimes are dynamic and require the application of quantitative modeling of systems biology and electrophysiology. Moreover, recent QTL mapping has also discovered large-effect alleles, including Saltol and *qSKC1*, but there is a slow rate of introgression into elite cultivars because of interactions between genotype and environment, and lack of knowledge about causal variants. The achievement of next-generation mapping with high-density GWAS panels, pan-genome resources and NGS-based bulk segregant analysis (BSA) can speed up the process of identifying loci that are stable and independent of the environment. An equivalent priority is to functionally validate QTL candidates on CRISPR/Cas-mediated knockout, knock-in, and base-editing systems.

Beyond bulk proteomic approaches such as iTRAQ and LC–MS/MS, emerging technologies including single-cell proteomics and spatial proteomics offer promising opportunities to resolve cell-type-specific and tissue-context-dependent protein dynamics under salt stress. These approaches will enable precise mapping of proteome heterogeneity within roots and shoots, thereby improving understanding of localized stress perception, signaling, and adaptive responses.

It will be necessary to integrate transcriptomic, proteomic, metabolomic and phenomic data that will produce predictive models of salt tolerance that reflect multilayer regulatory interactions. Combining these methods with high-throughput field phenotyping and machine learning will allow the breeding choice to be made more accurately. Besides this, rice wild relatives, halophytes and landraces are yet to be tapped and should be exploited methodically through multi-omics and haplotype-based association approaches. Altogether, the further studies are to focus on the translational pipelines, which will be able to bridge the gap between the molecular findings and the breeding programs and help to create the resilient types that would be able to endure the increasing salinities pressure caused by the climate change.

### 8.1 Key challenges and future development trends in rice salt stress research

Despite substantial advances in understanding rice salt stress responses, several key challenges remain. One major limitation is the incomplete functional validation of salt-responsive genes and proteins identified through omics approaches, particularly under field-relevant conditions. Many regulatory components exhibit strong developmental stage specificity and genotype-by-environment interactions, complicating their direct deployment in breeding programs.

Another challenge lies in resolving the spatial and temporal regulation of stress signaling, especially the coordination between Ca²^+^ signaling, ROS dynamics, and intracellular ion transport across different tissues and subcellular compartments. Integrating organelle-level proteomics, redox metabolomics, and high-resolution imaging will be critical for dissecting these processes. In addition, translating GWAS and QTL discoveries into elite cultivars remains slow due to limited knowledge of causal variants and regulatory mechanisms.

Future research is therefore expected to focus on integrative multi-omics frameworks combined with genome editing, quantitative modeling, and high-throughput field phenotyping. Such approaches will be essential for bridging the gap between molecular insights and practical breeding outcomes, ultimately enabling the development of rice cultivars with durable salt tolerance under changing climatic conditions.

## 9 Conclusion

Salt stress remains among the worst abiotic restrictions to rice productivity in most parts of the world that influences growth, metabolism, ion homeostasis, and yield at almost all stages of development. There has been considerable advancement in the understanding of physiological, molecular and genetic blueprints of salinity tolerance. The proteomic analysis demonstrates a massive cellular system restructuring, such as cellular signaling pathways, ROS detoxification, carbohydrate and energy metabolism, cytoskeleton formation, and ion transport machinery, suggesting that salt tolerance is regulated by a very complex, multi-layered, adaptive processes. The development of QTL mapping and molecular genetics has found key loci including Saltol, *qSKC1* and a number of new candidate regions that provide useful access points to breeding via marker-assisted and genomic technologies.

Although these have been achieved, most salt-responsive genes, proteins, and regulatory pathways have not been well characterized, which constrains the ability to translate the simple findings into sound cultivars. Proteomics combined with transcriptomics combined with metabolomics combined with genome editing has started to bridge this gap, and opportunities are available to distinguish the causal genes, authenticate their functions and pyramid advantageous alleles. CRISPR/Cas technologies, especially, have shown good potential of producing a specific set of changes leading to an enhanced ion balance, antioxidative capacity, and root system architecture under salinity.

In general, the combination of multi-omics applications, the development of sophisticated breeding practices, and genome engineering has become an effective platform to hasten the production of salt-tolerant rice strains. These innovations will play a critical role in protecting the rice production and enhancing agricultural sustainability as well as ensuring the availability of food to the future generation as soil salinization increases all over the world due to climate change.
