# Supplementary material for: Regulatory networks and molecular mechanisms underlying salt stress tolerance in rice
Source: Front Plant Sci. 2026 Mar 5;17:1757448. doi: 10.3389/fpls.2026.1757448 (PMC12999462; doi:10.3389/fpls.2026.1757448)
Supplement: Supplementary file 1 [file Table1.docx]

Table S1. Candidate proteins are involved in salt stress tolerance process in rice leaves.

| **Gene Symbol** | **Protein** | **Response** | **Functional Category** | **Reference** |
| --- | --- | --- | --- | --- |
| **RPK** | Receptor-like protein kinase | U | Salt perception & signal transduction | (Cheng et al., 2009) |
| **GEP2** | Guanine nucleotide-exchange factor 2 | D | Salt perception & signal transduction | (Chitteti & Peng, 2007) |
| **CaBP** | Putative calcium-binding protein | U | Salt perception & signal transduction | (Li et al., 2010) |
| **CRT** | Calreticulin | D | Salt perception & signal transduction | (Li et al., 2010) |
| **CRT** | Calreticulin | U | Salt perception & signal transduction | (Nam et al., 2012) |
| **CRT** | Calreticulin precursor | D | Salt perception & signal transduction | (Li et al., 2010) |
| **POD** | Peroxidase | U | ROS detoxification & defense | (Nam et al., 2012) |
| **CAT** | Catalase | U | ROS detoxification & defense | (Li et al., 2010) |
| **GR** | Glutathione reductase | U | ROS detoxification & defense | (Li et al., 2010) |
| **GlyI** | Glyoxalase I | U | ROS detoxification & defense | (Li et al., 2010) |
| **GlyII** | Glyoxalase II | U | ROS detoxification & defense | (Nam et al., 2012) |
| **POD1** | Peroxidase 1 precursor | U | ROS detoxification & defense | (Cheng et al., 2009) |
| **GST-II** | Glutathione S-transferase II | U | ROS detoxification & defense | (Nam et al., 2012) |
| **ANX** | Annexin-like protein | U | Membrane processes & transport | (Li et al., 2010) |
| **ACT7** | Actin 7 | U | Cell-wall structure & modification | (Chitteti & Peng, 2007) |
| **ACT** | Actin | U | Cell-wall structure & modification | (Li et al., 2010) |
| **β-Tub** | β-tubulin | D | Cell-wall structure & modification | (Li et al., 2011) |
| **COMT** | Caffeic acid 3-O-methyltransferase | D | Cell-wall structure & modification | (Li et al., 2010) |
| **CCoAOMT** | Caffeoyl-CoA O-methyltransferase | D | Cell-wall structure & modification | (Li et al., 2010) |
| **SAMS2** | S-adenosylmethionine synthetase 2 | D | Cell-wall structure & modification | (Li et al., 2010) |
| **SAMS** | S-adenosylmethionine synthetase | D | Cell-wall structure & modification | (Li et al., 2010) |
| **HSP90** | Heat shock protein 90 | U | Protein synthesis, folding & processing | (Li et al., 2010) |
| **DnaK** | DnaK-type chaperone precursor | U | Protein synthesis, folding & processing | (Nam et al., 2012) |
| **CPN60-1** | Mitochondrial chaperonin CPN60-1 | U | Protein synthesis, folding & processing | (Nam et al., 2012) |

Note: U= Upregulated, D= Downregulated

Table S2. Candidate proteins are involved in salt stress tolerance process in rice roots.

| **Gene Symbol** | **Protein** | **Response** | **Functional Category** | **Reference** |
| --- | --- | --- | --- | --- |
| **GPB-LR** | G-protein β-subunit-like protein | D | Salt perception & signal transduction | (Li et al., 2011) |
| **GTP** | Putative GTP-binding protein | D | Salt perception & signal transduction | (Li et al., 2011) |
| **CRT** | Calreticulin precursor | U | Salt perception & signal transduction | (Li et al., 2011) |
| **NDPK** | Nucleoside diphosphate kinase | U | Salt perception & signal transduction | (Li et al., 2011) |
| **Cu-Zn SOD** | Cu/Zn SOD precursor (chloroplast) | U | ROS detoxification & defense | (Li et al., 2011) |
| **APX** | Ascorbate peroxidase | U | ROS detoxification & defense | (Li et al., 2011) |
| **DHAR** | Dehydroascorbate reductase | U | ROS detoxification & defense | (Li et al., 2011) |
| **H-ATP** | Putative H⁺-transporting ATP synthase | D | Membrane processes & transport | (Li et al., 2011) |
| **PAL** | Phenylalanine ammonia-lyase | U | Cell-wall structure & modification | (Li et al., 2011) |
| **SAMS** | S-adenosylmethionine synthetase | U | Cell-wall structure & modification | (Li et al., 2011) |
| **EF-Tu** | Elongation factor Tu | D | Protein synthesis, folding & processing | (Li et al., 2011) |
| **Rbp** | mRNA-binding protein precursor | U | Protein synthesis, folding & processing | (Li et al., 2011) |
| **PDI** | Protein disulfide isomerase | U | Protein synthesis, folding & processing | (Li et al., 2011) |
| **CPN60A** | Chaperonin 60 α | U | Protein synthesis, folding & processing | (Li et al., 2011) |
| **CPN60B** | Chaperonin 60 β precursor | U | Protein synthesis, folding & processing | (Li et al., 2011) |
| **rbcL** | Rubisco large subunit | D | Photosynthesis & carbon assimilation | (Li et al., 2011) |
| **rbcL** | Rubisco large chain | D | Photosynthesis & carbon assimilation | (Liu et al., 2014) |
| **rbcC** | Rubisco small chain C | D | Photosynthesis & carbon assimilation | (Wei et al., 2021) |
| **CAB** | Chlorophyll a/b-binding protein | U | Photosynthesis & carbon assimilation | (Wei et al., 2021) |
| **CAB-8** | Chlorophyll a/b-binding protein 8 | D | Photosynthesis & carbon assimilation | (Wei et al., 2021) |
| **atpB** | Chloroplastic ATP synthase β | U | Photosynthesis & carbon assimilation | (Wei et al., 2021) |
| **HCF136** | PSII assembly/stability factor | U | Photosynthesis & carbon assimilation | (Li et al., 2011) |
| **ATP-B** | ATP-B gene product | D | Photosynthesis & carbon assimilation | (Li et al., 2011) |
| **ATP5B** | Mitochondrial ATP synthase β-subunit | D | Photosynthesis & carbon assimilation | (Liu et al., 2014) |
| **PGK** | Phosphoglycerate kinase | D | Carbohydrate & energy metabolism | (Wei et al., 2021) |
| **PMM** | Phosphomannomutase | D | Carbohydrate & energy metabolism | (Wei et al., 2021) |
| **TKL** | Transketolase | D | Carbohydrate & energy metabolism | (Nam et al., 2012) |
| **PGM** | Phosphoglycerate mutase | D | Carbohydrate & energy metabolism | (Wei et al., 2021) |
| **ENO** | Enolase | D | Carbohydrate & energy metabolism | (Nam et al., 2012) |
| **FRK1** | Fructokinase 1 | D | Carbohydrate & energy metabolism | (Wei et al., 2021) |
| **GPDH** | Glyceraldehyde-3-phosphate dehydrogenase | D | Carbohydrate & energy metabolism | (Nam et al., 2012) |
| **CS** | Cysteine synthase | D | Carbohydrate & energy metabolism | (Wei et al., 2021) |
| **IMDH3** | 3-Isopropylmalate dehydrogenase | U | Carbohydrate & energy metabolism | (Wei et al., 2021) |
| **GGPPS** | Geranylgeranyl diphosphate synthase | D | Carbohydrate & energy metabolism | (Wei et al., 2021) |

Note: U= Upregulated, D= Downregulated

Table S3: Candidate proteins are involved in salt stress tolerance process in rice plasma membrane.

| **Gene Symbol** | **Protein** | **Response** | **Functional Category** | **Reference** |
| --- | --- | --- | --- | --- |
| **CaM** | Calmodulin | D | Salt perception and signal transduction | (Cheng et al., 2009) |
| **DREEP** | Developmentally regulated plasma-membrane polypeptide | U | Salt perception and signal transduction | (Cheng et al., 2009) |
| **V-ATPaseE** | V-type H⁺-ATPase subunit E | U | Membrane transport and ion homeostasis | (Cheng et al., 2009) |
| **REM1** | Putative remorin 1 protein | U | Membrane structure and signaling | (Cheng et al., 2009) |

Note: U= Upregulated, D= Downregulated

Table S4. Mapping Populations and Marker Density

| **Mapping Population** | **Population Type** | **Total Markers** | **Marker Type** | **Reference** |
| --- | --- | --- | --- | --- |
| Nonabokra × Koshihikari 133 F2 | F2 | 161 | RFLP | (Lin et al., 2004) |
| Pokkali × IR29 78 RILs | RIL | 23 | SSR | (Bonilla et al., 2002; Thomson et al., 2010) |
| Pokkali × IR29 140 RILs | RIL | 100 | SSR | (Thomson et al., 2010) |
| Nona Bokra × Koshihikari 192 BC2F2 / 2973 BC3F3 NILs | BC2F2 / BC3F3 NIL | 14 | AFLP/SST | (Genc et al., 2007) |
| IR59462/Nona Bokra/Pokkali//IR4630-22-2-5-1-3/IR10167-129-3-4 150 F7 NILs | NIL | 4 | — | (Flowers et al., 2000) |
| IR4630/IR15324 118 RILs | RIL | — | AFLP/RFLP | (Koyama et al., 2001) |
| CSR27/MI48 216 F2/F3 RILs | RIL | — | SSR | (Ammar et al., 2009) |
| CSR27/MI48 216 F7 RILs | RIL | 1058 | SSR | (Pandit et al., 2010) |
| Zaiyeqing8/Jingxi17 DH | DH | — | — | (Gong et al., 1999) |
| Sadri/FL478 232 F2 | F2 | 155 | SSR | (Mohammadi et al., 2013) |
| Cheriviruppu/Pusa Basmati 1 218 F2/F3 | F2/F3 | 131 | SSR | (Hossain et al., 2015) |
| Kalarata × Azucena 400 F2 | F2 | 151 | SSR | (de Ocampo et al., 2022) |
| CSR10/PS5 140 F2 | F2 | 100 | HvSSR | (Pundir et al., 2021) |
| Wujiaozhan × Nipponbare 181 BC1F2 | BC1F2 | 157 | SSR | (Zeng et al., 2021) |
| CSR11/MI48 208 | — | 6,068 | SNP | (Tiwari et al., 2016) |
| Weiguo × IR36 199 F2:3 | F2:3 | — | KASP | (Lei et al., 2020) |
| MAGIC population 221 DC1 | MAGIC | — | 55k SNP array | (Zhang et al., 2020) |
| IR-44595 × IR-318 168 F2 | F2 | 2221 | SNP | (Goto et al., 2022) |
| Pokkali × IR29 80 RILs | RIL | 206 | — | (Gregorio et al., 2013) |
| Pokkali × IR29 181 BC3F4 | BC3F4 | 40 | SSR | (Rahman et al., 2017) |
| Milyang23 × Gihobyeo 164 F18:F19 RILs | RIL | 1300 | RFLP/SSLP/AFLP/Isozyme | (Takehisa et al., 2004) |
| Tarommhalli × Khazar 192 F2/F3 | F2/F3 | 74 | SSR | (Lee et al., 2007) |
| IR26 × Jiucaiqing 150 F2:9 RILs | RIL | — | — | (Sabouri & Sabouri, 2008) |
| Jiucaiqing × IR26 150 F2:9 RILs | RIL | 135 | SSR | (Wang et al., 2011) |
| Gharib × Sepidroud 148 F2:4 | F2:4 | 131 | SSR/AFLP | (Wang et al., 2012) |
| Hasawi × BRR dhan28 435 BC1F2 | BC1F2 | 6209 | SNP | (Ghomi et al., 2013) |
| Horkuch × IR29 137 F2:3 | F2:3 | 2230 | SNP | (Mondal et al., 2022) |
| Akundi × BRRI dhan49 F2:3 | F2:3 | 884 | SNP | (Maniruzzaman et al., 2022) |
